# Novel *Bartonella* Agent as Cause of Verruga Peruana

**DOI:** 10.3201/eid1907.121718

**Published:** 2013-07

**Authors:** David L. Blazes, Kristin Mullins, Bonnie L. Smoak, Ju Jiang, Enrique Canal, Nelson Solorzano, Eric Hall, Rina Meza, Ciro Maguina, Todd Myers, Allen L. Richards, Larry Laughlin

**Affiliations:** Uniformed Services University of the Health Sciences, Bethesda, Maryland, USA (D.L. Blazes, K. Mullins, B.L. Smoak, L. Laughlin);; Walter Reed Army Institute of Research, Silver Spring, Maryland, USA (B.L. Smoak);; Naval Medical Research Center, Silver Spring (J. Jiang, E. Hall, T. Myers, A.L. Richards);; Naval Medical Research Unit 6, Lima, Peru (E. Canal, R. Meza);; Hospital San Juan de Dios, Lima (N. Solorzano);; Universidad Peruana Cayetano Heredia–Tropicales, Lima (C. Maguina)

**Keywords:** Bartonellosis, emerging infection, C*andidatus* Bartonella ancashi, Peru, pathogen discovery, bacteria, verruga peruana, vector-borne infections, Bartonella

## Abstract

While studying chronic verruga peruana infections in Peru from 2003, we isolated a novel *Bartonella* agent, which we propose be named *Candidatus* Bartonella ancashi. This case reveals the inherent weakness of relying solely on clinical syndromes for diagnosis and underscores the need for a new diagnostic paradigm in developing settings.

Bartonellosis is a disease caused by infection with species from the *Bartonella* genus. In South America, infection with *B. bacilliformis*, an α-2 proteobacterium, may cause a life- threatening bacterial infection (*1*,*2*). If untreated, the acute form of the illness, sometimes referred to as Oroya fever, has a high mortality rate because the bacteria invade erythrocytes, resulting in subsequent severe anemia and secondary infections. A chronic phase, termed verruga peruana, is characterized by vasculoproliferative skin lesions; some reseachers have also described an asymptomatic bacteremic phase, which may contribute to the longevity of the reservoir status of infected persons (*3*).

In 2007, a novel species of *Bartonella* (*B. rochalimae*) was isolated from a single traveler who had an acute febrile anemia after traveling to Peru (*4*). We report the identification of another novel agent of *Bartonella* isolated from a patient with chronic bartonellosis (collected in 2003, fully characterized in 2011–2012). We suggest that the isolate be named *Candidatus* Bartonella ancashi in honor of the highland region of Peru.

## The Study

**Figure 1:**
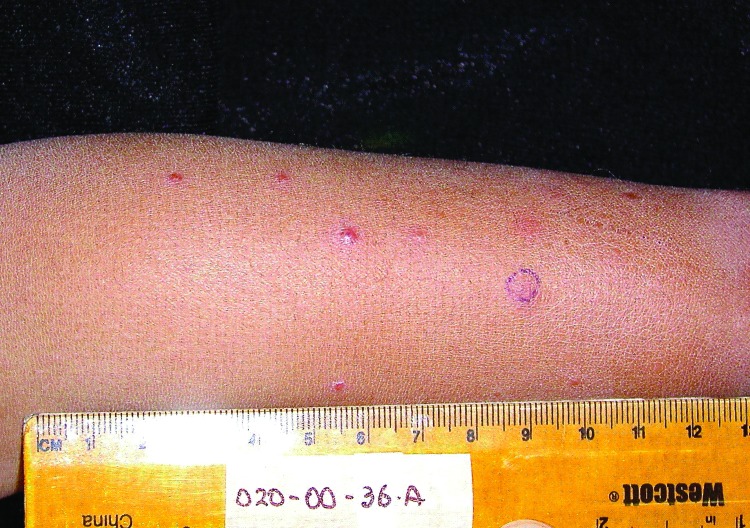
Clinical presentation of verruga peruana in 3-year-old boy, Peru, 2003.

The patient was a 3-year-old boy with no known underlying medical history who was identified in 2003 as having clinical verruga peruana by the classic appearance of the eruptive nodular rash (Figure 1). He and his family lived in a rural setting near the town of Caraz, Ancash region, Peru. He lived in close proximity to numerous pets and farm animals and had experienced insect bites around the time the eruptive rash developed. His rash had been present for ≈30 days, and he had no fevers, chills, or arthralgias. Baseline laboratory studies included complete blood counts and bacterial culture for *Bartonella* species, using methods previously described (*5*,*6*). Briefly, the media was biphasic, consisting of Bacto agar with Proteose Peptone No. 3 (Becton, Dickinson and Co., Sparks, MD, USA), dextrose, sodium chloride and 10% defibrinated sheep’s blood, and RPMI supplemented with 10% inactivated fetal bovine serum.

The physical examination revealed that the child had 56 lesions, distributed mainly on the extremities. Laboratory values were the following: hemoglobin level 12.8 mg/dL (reference range 11–13 g/dL), hematocrit 39% (reference range 31%–43%), and platelet count of 300,000/µL (reference range 15,000–400,000); his leukocyte count was elevated at 28,000/µL (reference range 4,100–10,900) with 51% eosinophils, for which he was referred for further evaluation. 

The peripheral blood smear was negative for intracellular organisms, but blood culture was positive for a *Bartonella* species. This species was further studied and found to be novel on the basis of genetic sequencing of the isolate obtained from the standard blood culture (*7*). His condition was treated with azithromycin, and the rash fully resolved (*8*,*9*). To confirm the identity of the isolate from this patient, in 2011–2012 we conducted molecular analyses (including PCR, nested PCR [nPCR], and sequencing) on the whole blood culture isolate, *Bartonella* species no. 20.00 (*10*).The isolate was characterized by sequencing 3 gene fragments.

The following conditions were used for PCR amplification. For *rrs*, initial denaturation was at 95°C for 1 min, followed by 45 cycles of denaturation at 95°C for 30 s, annealing at 56°C for 30 s, and elongation at 68°C for 90 s for PCR and 70 s for nPCR, and then by a final extension step at 72°C for 7 min. For *gltA*—95°C, denaturation was for 1 min, followed by 45 cycles of denaturation at 95°C for 30 s, annealing at 51°C for 30 s, and elongation at 68°C for 60 s, and then by the final extension step at 72°C for 7 min. For *rpoB*, primers were selected from the conserved regions of RNA polymerase β-subunit encoding gene (*rpoB*) after alignment of the *rpoB* from *B. quintana* and *B. vinsonni* for PCR and nPCR. PCR and nPCR were carried out by using conditions identical to those described for *gltA* (Table).

**Table:** Primers used for PCR, nested PCR, and sequencing of novel *Bartonella* isolate from Peru, 2011–2012*

Gene	Primer name	Primer sequence, 5’ → 3’	Use	Fragment length
*rrs*	16SU17F	AGAGTTTGATCCTGGCTCAG	PCR, nPCR, sequencing	1,424 bp
	16SU1592R	AGGAGGTRATCCAGCCGCA	PCR, nPCR, sequencing	
	16SU 833R	CTACCAGGGTATCTAATCCTGTT	nPCR, sequencing	
	16S E. coli-518F	CAGCAGCCGCGGTAATAC	nPCR, sequencing	
*gltA*†	BHCS 781p (F)	GGGACCAGCTCATGGTGG	PCR, sequencing	338 bp
	BHCS 1137n (R)	AATGCAAAAAGAACAGTAAACA	PCR, sequencing	
*rpoB*	BrpoB1435F	CGCATTGGTTTRCTTCGTATG	PCR	589 bp
	Brpo2327R	GTAGACTGATTAGAACGCTG	PCR, nPCR, sequencing	
	Brpo1696F	CCTACGCATTATGGTCGTATTTG	nPCR, sequencing	

*nPCR, nested PCR. †*gltA* primers were previously described by Eremeeva et al. (*4*).

PCR products were purified by using the QIAquick PCR Purification Kit (QIAGEN, Valencia, CA, USA) before sequencing. PCR products were sequenced in both directions by using the BigDye Terminator v3.1 Cycle Sequencing Kit (Life Technologies, Carlsbad, CA, USA) and run on an automated 3130*xl* Gene Analyzer (Life Technologies). Sequences were characterized by BLAST analysis (http://blast.ncbi.nlm.nih.gov/Blast.cgi). The sequence analysis was performed by using BioEdit version 7.1.3 (Ibis Biosciences, Carlsbad, CA, USA). The multiple sequence alignments were performed with the ClustalW multiple alignment application also in BioEdit version 7.1.3. Phylogenetic trees were created with MEGA5 software using the neighbor-joining tree method with 1,000 bootstrap replicates (*11*).

**Figure 2:**
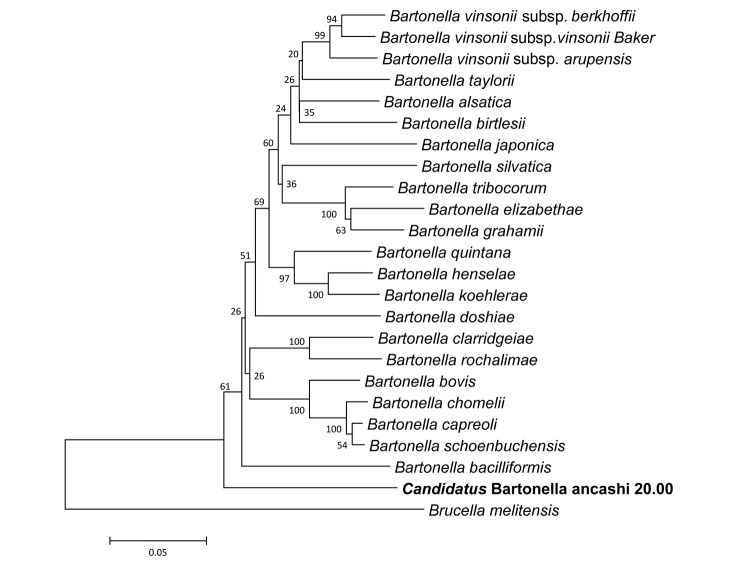
Phylogeny for concatenated sequences of novel *Bartonella* isolate (**boldface**), including a 312-character fragment of *gltA* and a 589-character fragment of *rpoB*. The neighbor-joining tree method (1,000 bootstrap replicates) was employed using MEGA5 software (*11*), and the distances were calculated by using the Jukes-Cantor method, in which units are calculated as the number of base pair substitutions per site (*10*). *Brucella melitensis* was used as the outgroup.

Subsequent comparison with known *Bartonella* species in GenBank found no identical sequences. The 1,351-bp sequence of the *rrs* fragment was found to be 99.0% similar to the *rrs* fragment of *B. bacilliformis.* The 312- and 589-bp fragments of *gltA* and *rpoB*, respectively, were found to be most similar to their counterparts of *B. bovis* at 89.4% (*gltA*) and 85.9% (*rpoB*), respectively (Technical Appendix). The sequence similarity ranges for the *rrs*, *gltA*, and *rpoB* for recognized *Bartonella* species are 97.7%–99.8%, 83.4%–96.1%, and 85.9%– 96%, respectively (*12*). In addition, *rpoB* and *gltA* are believed to have the best discriminating power for *Bartonella* species (*13*). La Scola et al. proposed that a new species be designated if the sequence similarities are <96% and <95.4% for a 327-bp fragment of *gltA* and a 825-bp fragment of *rpoB*, respectively (*13*). The sequence similarities for *Candidatus* Bartonella ancashi 20.00 to other known *Bartonella* species fall well below these suggested values, providing more evidence that this agent is unique. Phylogenetic analysis of the *rrs*, *gltA,* and *rpoB*, gene fragments provide additional evidence for identification of a unique *Bartonella* agent. The concatenated sequence of *gltA* and *rpoB* gene fragments placed the new *Bartonella* isolate in an exclusive clade that is most closely aligned with *B. bacilliformis* (Figure 2). The *rrs* fragment also placed the isolate in a clade with *B. bacilliformis*. The results from the phylogenetic analysis combined with the sequence similarity data provide evidence that this isolate, *Candidatus* Bartonella ancashi 20.00, is unique (*12*,*13*).

## Conclusions

This case underscores the inherent weakness of relying solely on clinical syndromes for diagnosis. The variety of bacteria that have been implicated in the clinical spectrum of bartonellosis is increasing as molecular methods are applied to isolates that previously were identified by using clinical criteria or biochemical testing. The novel bacterium may have similar epidemiologic, clinical, and microbiologic properties to *B. bacilliformis*, but without relating these data to a full molecular characterization, that assumption is precarious.

To address this public health deficiency, a new diagnostic paradigm should be deployed to developing settings such as Peru. This is particularly true for areas with high biodiversity, a point identified by other investigators who have termed these regions “hot zones” for emerging infectious diseases (*14*). Unfortunately, tools such as high-throughput sequencing are rare in developing settings where risk for novel pathogen emergence is highest. Investment in advanced molecular diagnostic platforms in the developing setting will be an essential tool for expanding pathogen discovery; of course, this should be accompanied by parallel investments in training in molecular laboratory techniques and analysis for resident scientists. Opportunities for grants and stable faculty positions must also be supported to encourage qualified scientists to remain in the developing setting.

Finally, evidence indicates that humans contract bartonellosis only once and that lifelong immunity results from that primary infection (*15*). Because of this circumstance, and the inability to identify an animal reservoir of *B. bacilliformis*, Peruvian scientists and others have identified bartonellosis as a disease that may be eradicated in the Andean region through development of a vaccine against *B. bacilliformis*, targeted treatment of patients, and vector control programs (*15*). This possibility may be less feasible if multiple species of *Bartonella* cause bartonellosis. Further molecular and immunologic studies should be undertaken if this disease is to be targeted for eradication.

Technical Appendix.  Comparison of rpoB and gltA sequence similarities for Bartonella species. The 312- and 589-bp fragments of gltA and rpoB, respectively, were found to be most similar to their counterparts of Bartonella bovis at 89.4% (gltA) and 85.9% (rpoB).
