# Supplementary material for: Novel Bartonella Agent as Cause of Verruga Peruana
Source: Emerg Infect Dis. 2013 Jul;19(7):1111–4. doi: 10.3201/eid1907.121718 (PMC3713980; doi:10.3201/eid1907.121718)
Supplement: Supplementary file 1 — Technical Appendix. Comparison of rpoB and gltA sequence similarities for Bartonella species. The 312- and 589-bp fragments of gltA and rpoB, respectively, were found to be most similar to their counterparts of Bartonella bovis at 89.4% (gltA) and 85.9% (rpoB). [file 12-1718-Techapp-s1.pdf]

Article DOI: http://dx.doi.org/10.3201/eid1907.121718

Novel *Bartonella* Agent as Cause of Verruga Peruana

Technical Appendix

Technical Appendix Table. *rpoB* (top) and *gltA* (bottom) sequence similarities for *Bartonella* species

	Percentage similarity with taxon																							
Taxon	1	2	3	4	5	6	7	8	9	10	11	12	13	14	15	16	17	18	19	20	21	22	**23**	24
1) *B. alsatica*		88.2	90.5	87	86.8	87	88.7	87.9	88.6	88.7	91	91.7	90.3	91.5	87.3	87	90.1	91.9	90	92.8	92.8	92.6	**84**	73.2
2) *B. bacilliformis*	84.9		86.8	88.2	88.4	87.7	88	86.8	84.2	84.5	88.2	86.8	87.2	87.7	87	88.2	85.8	87.3	85.8	87.2	85.9	86.5	**84.9**	71.9
3) *B. birtlesii*	92.9	85.8		85.9	86.5	85.9	86.5	87.3	86.1	86.6	88.4	89.4	87.7	88.6	85.1	86.6	87.5	89.4	88.9	91	90.3	91.4	**83.3**	72.5
4) *B. bovis*	87.8	87.1	90		94.7	94.3	87.3	89.3	84.9	85.6	88.2	86.3	89.3	87.5	87.2	94.9	86.6	86.5	86.1	86.5	86.1	87.2	**85.9**	73.3
5) *B. capreoli*	88.1	87.8	88.4	94.8		97.8	87.3	89.3	84.5	85.2	88.6	87	88.4	88	87.3	99.8	86.8	87.2	86.3	86.8	86.5	87.5	**85.2**	73.2
6) *B. chromelii*	87.1	85.5	88.1	95.1	97.1		87.5	88.7	84.7	85.1	87.9	87.2	88	87.7	87.2	98	86.6	86.3	86.1	86.8	87	87.3	**84.7**	73.5
7) *B. clarridgeiae*	86.2	85.2	87.8	90.7	90.3	89.4		87.2	85.1	85.6	87.5	87.2	87.9	88.4	91.7	87.5	85.1	87.9	86.5	88	87.3	87.5	**84.5**	71.9
8) *B. doshiae*	87.1	84.6	89.4	87.8	86.8	87.1	87.8		85.1	87.2	90.3	87	90.7	89.3	86.8	89.1	85.9	88.7	87.5	88	88	88.4	**85.2**	73
9) *B. elizabethae*	87.8	83.9	89.1	88.4	88.7	89.1	87.8	86.8		93.1	87	86.1	87.9	87	84.4	84.7	88.2	87.3	93.6	87	87.5	86.8	**82.4**	73.2
10) *B. grahamii*	89.4	85.8	91	90.7	90.7	91	88.1	88.1	94.8		87.9	86.8	88.2	87.9	84.4	85.4	87.5	87.3	93.6	87.9	87.7	87.7	**82.4**	74.6
11) *B. henselae*	89.4	84.2	91	90.7	87.5	88.1	87.1	87.1	86.5	89.1		88.6	95.6	93.3	86.6	88.7	87.2	89.4	88.9	89.3	89.3	90	**84**	73.7
12) *B. japonica*	85.2	83	87.1	85.5	85.8	84.9	85.5	83.9	84.6	87.1	87.1		88	88.9	85.4	87.2	89.3	88.9	89.1	91.4	91.2	91.2	**84.4**	72.3
13) *B. koehlerae*	89.7	84.9	91	89.4	87.8	88.1	87.8	85.5	87.8	89.1	94.5	86.8		91.5	86.1	88.6	86.8	89.4	88.7	88.4	88.7	89.1	**83.8**	72.8
14) *B. quintana*	88.7	83.9	88.7	89.4	88.7	87.5	88.7	85.5	85.8	87.8	92.3	87.5	91.3		87.3	88.2	87	90.8	88.6	91.2	91	91.4	**84.5**	74.7
15) *B. rochalimae*	86.2	86.2	88.7	91.9	91.6	90.7	96.1	86.5	88.4	89.4	89.1	85.5	87.8	89.1		87.2	84	85.8	85.8	85.9	85.2	85.6	**84.7**	72.5
16) *B. schoenbuchensis*	86.8	85.5	87.8	94.5	97.1	99.3	89.4	86.8	89.1	91	87.5	84.6	87.5	87.5	90.7		87	87.3	86.5	87	86.6	87.7	**85.1**	73.3
17) *B. silvatica*	85.8	82	86.5	86.5	87.1	86.8	88.7	85.5	86.2	89.1	85.8	84.2	85.2	87.5	88.4	87.1		88.6	87.7	89.3	88.9	88.9	**83.7**	72.8
18) *B. taylorii*	90.7	85.5	91.3	88.7	89.1	88.1	87.1	86.8	87.8	89.7	89.4	87.8	89.4	89.7	88.4	87.8	90.3		88	91.4	90.7	91.7	**82.8**	73.7
19) *B. tribocorum*	90.7	86.8	91.9	90	89.1	88.4	88.1	87.1	94.5	95.8	89.4	85.5	89.7	88.1	90	88.4	87.1	90		88.2	88.7	89.3	**83.3**	72.6
20) *B. vinsonii* subsp. *arupensis*	91.9	84.6	91.6	90	89.1	90	88.4	89.1	90.3	91.6	90.3	89.1	90	90.7	89.1	89.7	88.7	92.9	91		94.7	95.2	**83.5**	73.7
21) *B. vinsonii* subsp. *berkhoffii*	92.6	85.2	91.6	89.7	87.8	88.4	88.1	87.5	89.4	90.7	90.7	86.8	89.4	89.4	88.7	88.1	88.7	92.3	91.3	94.8		96.1	**82.4**	74.4
22) *B. vinsonii* subsp. *vinsonii*	92.9	86.5	91.6	89.1	89.1	89.7	88.1	88.4	90.3	91.3	90	87.8	90	89.4	89.4	89.4	89.1	94.5	91.3	95.8	96.1		**84**	74
**23) Candidatus B. ancashi 20.00**	**86.5**	**84.9**	**88.1**	**89.4**	**87.1**	**86.8**	**84.6**	**85.5**	**84.9**	**83.9**	**87.1**	**81.7**	**85.8**	**84.9**	**85.5**	**86.5**	**81**	**84.9**	**85.5**	**85.5**	**85.2**	**85.8**		73.3
24) *Brucella melitensis*	69.8	70.1	67.9	69.8	71.7	70.5	69.2	67.3	70.8	69.8	69.2	69.8	68.2	69.5	69.8	70.5	69.5	67.9	69.5	70.5	71.4	68.5	**68.2**	

*Similarities are based on a 312-bp fragment of *gltA* and a 589- bp fragment of *rpoB.* **Boldface** indicates similarities of the novel isolate.
